# Snoozing through the storm: torpor use during a natural disaster

**DOI:** 10.1038/srep11243

**Published:** 2015-06-15

**Authors:** Julia Nowack, A. Daniella Rojas, Gerhard Körtner, Fritz Geiser

**Affiliations:** 1 Centre for Behavioural and Physiological Ecology, Zoology, University of New England, Armidale NSW 2351, Australia

## Abstract

Although storms provide an extreme environmental challenge to organisms and are predicted to increase in frequency and intensity due to climate change, there are no quantitative observations on the behaviour and physiology of animals during natural disasters. We provide the first data on activity and thermal biology of a free-ranging, arboreal mammal during a storm with heavy rain and category 1 cyclone wind speeds. We studied a population of sugar gliders (*Petaurus breviceps*), a species vulnerable to bad weather due to their small body size and mode of locomotion, in a subtropical habitat during spring when storms are common. Although torpor is generally rare in this species, sugar gliders remained inactive or reduced foraging times during the storm and further minimized energy demands by entering deep torpor. All animals survived the storm and reverted to normal foraging activity during the following night(s). It thus appears that heterothermic mammals have a crucial adaptive advantage over homeothermic species as they can outlast challenging weather events, such as storms and floods, by reducing metabolism and thus energetic needs.

Anecdotes report that animals mysteriously disappear before and during storms and, if they survive, re-appear when the danger has passed. What do animals do during the storm and how do especially small mammals with high energy demands survive these challenging periods? Climatic disasters increasingly affect many parts of the world and can have adverse effects on humans and animals. Climatologists predict that changes in global weather patterns will increase the frequency and intensity of storms, floods, droughts and wild fires^1^,^2^. Unfortunately, few studies have focused on how animals respond to such challenges. Although there are reports of sharks escaping to the open sea prior to hurricanes^3^, elephants fleeing coastal regions during a tsunami^4^, or birds circumventing a tornadic storm^5^, the behaviour and physiology of animals during storms has never been quantified.

Recent studies have suggested that torpor use in heterothermic mammals, which is known as an effective adaptation of animals to survive seasonal food shortages and bad weather^6^, may also be of crucial importance to deal with unpredictable challenging conditions, such as unseasonal cold spells or famines^7^,^8^,^9^,^10^. Torpor allows animals to survive energy bottlenecks by reducing body temperature (T_b_) and energy expenditure^11^ and because torpid animals are usually concealed in a sheltered location^12^, they are protected from extreme environmental conditions and also predation^13^. Data on recent mammalian extinctions provide evidence that heterothermic species capable of employing torpor have a lower risk of extinction than homeothermic species that have to maintain a high T_b_ and energy expenditure and therefore have to continue foraging even under hostile conditions^14^.

The purpose of our study was to investigate how arboreal mammals, which are likely strongly affected by storms, adjust foraging and thermal biology during a storm. We studied a population of Australian sugar gliders (*Petaurus breviceps*,> ~130 g) inhabiting a coastal, subtropical habitat during spring, when the likelihood of storms is high. Other studies conducted on this species in a cool-temperate region observed torpor only occasionally when extremely cold and wet conditions substantially elevated their energy demands^15^,^16^. This small arboreal marsupial appears to be particularly vulnerable to storms since its mode of locomotion (gliding) is impeded by high wind speed. Furthermore, rain is known to reduce the amount of nectar^17^, which is one of the main food sources of sugar gliders besides insects, *Eucalyptus* sap and *Acacia* gum^18^. We therefore hypothesized that sugar gliders would limit foraging and enter torpor as a survival strategy during storms with high rainfall and wind.

## Results

### Ambient conditions

Daily average ambient temperature (T_a_) for the study period was 17.4 ± 2.8 °C, ranging from 6 °C at night to 37.5 °C during daytime. Average minimum and maximum T_a_ increased slightly from September to October (mean maximum T_a_ 23.1 ± 5.5 °C vs 24.7 ± 4.6 °C; mean minimum T_a_ 10.3 ± 2.4 °C vs 12.7 ± 2.1 °C). Wind speed for all nights of the study period (n = 43 days) averaged 17 ± 8 km/h and we recorded a total of 110.2 mm of nightly rainfall over six nights. 95 mm of this rain occurred during the night of the 14^th^ to 15^th^ of October, when the storm passed the area. During this night the maximum rainfall was 14.8 mm over 10 min, wind speed reached 94 km h^−1^ with an average of 54 km h^−1^ (the Bureau of Meteorology defines gusts between 90–125 km h^−1^ as category 1 cyclones) and minimum T_a_ was 9.5 °C.

### Activity & nesting sites

At daytime gliders nested solitarily or in groups of up to three individuals in tree hollows between 2.4 m to 13.8 m above ground. Gliders used *Angophora* or *Eucalyptus* trees (18.9 ± 6.2 m tree height) for nesting. Average nightly activity time was 464 ± 110 min. Activity usually started 95 ± 80 min after sunset and terminated 111 ± 73 min before sunrise. Duration of activity was significantly influenced by average wind speed (t_386_ = −4.02, p < 0.001, N = 402, n = 12), whereas T_a_ (t_386_ =−0.89, p = 0.84) and rainfall (t_386_ = −0.19, p = 0.35) had no significant effects. During the storm night activity was reduced in comparison to non-storm nights (average 129 ± 147 min, N = 10, n = 10, no data for two individuals, vs. 473 ± 94 min, N = 392, n = 12) and three individuals remained inactive in their nest (T_b_ < 38 °C); total inactivity was never recorded for any other night. Furthermore, six individuals showed reduced activity times between 40 min and 250 min and only one individual displayed a normal nightly activity of 470 min. During the storm individuals spent more time in close proximity to their tree hollow (78% to 100%) than they did on average (increase by 26–61% compared to the individuals’ average).

### Torpor use

**Figure 1:**
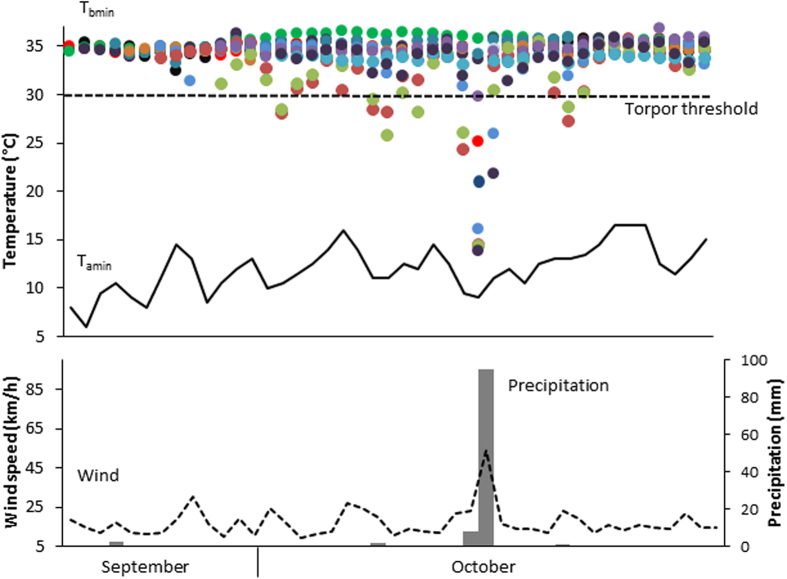
Individual minimum body temperature (T_bmin_; different colours indicate individuals) in relation to minimum ambient temperature (T_amin_; black line), precipitation (grey bars) and average wind speed (dashed line) per night.

**Figure 2:**
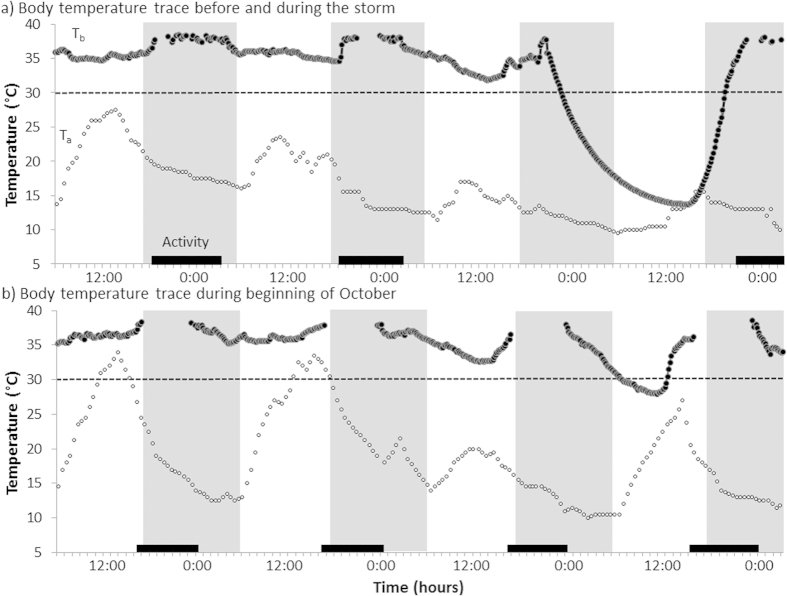
Body temperature traces (a) for a male sugar glider during three days before the storm and during the storm (12-15.10.14) and (b) a female individual for four days at the beginning of October (30.09—03.10.14). Body temperature (T_b_) is shown as black dots; ambient temperature (T_a_) as circles. The scotophase is indicated by grey bars and activity by black lines. The dashed line indicates the torpor threshold of 30 °C. Both figures show a torpor bout during the fourth day depicted.

Daily minimum T_b_ (T_bmin_) varied between 13.8 °C and 36.7 °C. The mean resting T_b_ (excluding torpor) was 34.5 ± 1.1 °C. Torpor was only observed on eight of 43 days. During non-storm days torpor was observed in either one or two individuals (N = 7, in six cases the same two females sharing a tree hollow) and T_b_ never decreased below 21.0 °C (range 21.7–29.4 °C; Fig. 1). In contrast, seven out of ten individuals for which data were available entered torpor during the storm. T_b_ during the storm reached 13.8 °C (Fig. 1) and average T_bmin_ during torpor was significantly lower than during any other torpor day (19.2 ± 6.3 °C, N = 7, n = 7 vs. 26.8 ± 2.3 °C, N = 11, n = 4; Wilcoxon rank test: W = 13, p = 0.020; Fig. 2). The second lowest individual T_bmin_ of 21.7 °C was observed on the day after the storm when two individuals entered torpor. After this day until the end of the study period 14 days later shallow torpor was expressed only once by the same two individuals that used torpor before the storm (Fig. 1). Entry into torpor during the first half of the night was only observed during the storm (1900 h–2200 h, N = 3), whereas on other days most torpor entries occurred between 0400 h and 0600 h, close to dawn. Moreover, torpor bout duration (TBD) was significantly shorter before and after the storm (mean 9.2 ± 2.2 h, maximum ~14 h, N = 11, n = 4) than during the storm (mean 12.6 ± 7.1 h, maximum ~23 h, N = 7, n = 7) (ANOVA, F_1, 10_ = 28.6, p = 0.003).

When we tested the number of torpid individuals/day for the impact of weather variables, we found a significant influence of an interaction between average wind speed and rainfall (r^2^ = 0.7237, df = 39, p = 0.027, N = 433, n = 12), whereas minimum T_a_ had no significant effect and hence was removed from the final model. In fact, torpor did only occur above a T_a_ of 9 °C and never on the coldest days.

### Estimated energy savings through torpor use

**Figure 3:**
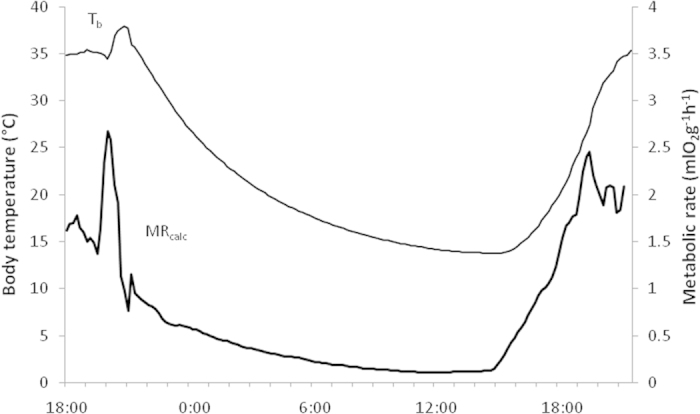
Body temperature (T_b_) and calculated metabolic rate (MR_calc_) of a torpor bout of a male sugar glider during the storm night.

To estimate the energetic significance of torpor use during the storm night, we calculated metabolic rates based on the continuous T_b_ measurements and daily average T_a_. Calculated metabolic rates indicated an energy saving of 9% during shallow torpor (TBD <4h, T_bmin_: 29.9 °C) and up to 67% during deep torpor (TBD ~ 23 h T_bmin:_ 14 °C; Fig. 3) compared to average metabolic rates during normothermia based on resting T_b_ averaged over at least two hours of the same night.

### Food availability

Of the five identified *Banksia* species (70 *B. integrifolia*,> 9 *B. ericifolia*,> 10 *B. serrata*,> 4 *B. spinulosa*,> 1 *B. oblongifolia)* only *B. ericifolia* and *B. integrifolia* flowered during the time of the study and the total number of inflorescences decreased over time from 41 to 7. Over the same period of time the abundance of trapped insects per night increased (range 0.05 g–3.89 g, N = 28). Insect abundance was correlated with minimum nightly T_a_ during sampling (r^2^ = 0.162, df = 26, p = 0.019; insect abundance (g) = 0.084T_a_ (°C)–0.157).

## Discussion

Our data show that sugar gliders reduced foraging and increased torpor use, depth and duration during a severe subtropical storm. Activity during the storm was significantly lower than during other nights, was mostly spent in close proximity to tree hollows, and torpor was used by most individuals. Interestingly, most animals recovered quickly from the impact of the storm and reverted to normal activity routine during the next night. Calculated metabolic rates indicate that the use of deep torpor during the storm allowed for extensive energy savings up to 67% compared with metabolic rates during rest and were in the same range as reported for laboratory measurements under similar conditions (saving of 60%; T_a_ = 11 °C; T_bmin_ = 13 °C; TBD:15 h)^19^. This suggests that the use of deep torpor would have compensated for most of the lost foraging opportunities during the severe weather condition.

Similarly, torpor during a blizzard has also been reported for pregnant hoary bats, *Lasiurus cinereus*. While this species used torpor when exposed to T_a_ ~ 5 °C during a snow storm in Canada^9^, torpor use during the spring storm in our study occurred at much warmer temperatures and appeared to be independent of T_a_. This is in contrast to previous studies on sugar gliders where torpor use was mainly triggered by cold and wet (T_a_ < 5 °C)^15^. However, because wind speed and rain were correlated with torpor occurrence our data suggest that the energy saving afforded by torpor allowed animals to remain inactive and to shelter during storm condition. For a gliding species inhabiting tree hollows as high as 14 meters above ground and usually foraging in the canopy, it will be impossible to move in their usual mode of locomotion during high wind speeds. In addition, individuals that were temporarily active during the storm night would have to cope with the intense rain, which would have further reduced gliding ability and also drastically elevated heat loss of these small animals^19^. Since some gliders stayed completely inactive during the storm, we conclude that gliders used torpor as a proactive strategy rather than as a direct response to elevated heat loss due to wet fur. Moreover, our data suggest that torpor was largely independent of food abundance, but rather used because foraging was impaired by bad weather. Although abundance of nectar was likely reduced during the storm^17^, *Banksia* inflorescence abundance was already low before the storm and animals likely met their energy demands by feeding on insects and tree exudates which are presumably less affected by wind and rain.

Cyclones are relatively common in tropical and subtropical habitats and can damage coastal as well as inland areas via gales and heavy rainfall^20^. Even after a cyclone has passed, or has decayed below cyclone strength, damage to vegetation and significant flooding may occur, which will affect access to food. Our data indicate that torpor may be important for survival of heterothermic species by allowing them to stay inactive and concealed during hostile conditions throughout the storm. This is especially important with regard to climate change. An increase of climatic disasters is anticipated in future^1^ and the intensity of storms will likely increase by 2–11% in the next 85 years^21^ and strongly affect tropical and subtropical regions and the animals living in them. In these conditions heterothermic species likely have a crucial adaptive advantage in comparison to homeothermic species as they can outlast challenging climatic periods as storm, floods and potentially other natural disasters^22^ by reducing metabolism and energy needs.

## Material and Methods

### Study site, ambient conditions and food abundance

The study was conducted in September-October 2014 (austral spring) at the Royal National Park located south of Sydney (Spring Gully Area; 34.09384 S, 151.13876 E). The methods were carried out in accordance with the approved guidelines and regulations for animal care at the University of New England. Approval to conduct this study was granted by the University of New England Animal Ethics Committee and New South Wales National Parks and Wildlife Service. We recorded T_a_ for every hour with a data logger (Thermochron iButton/DS1921G, Dallas Semiconductors; resolution 0.5 °C) placed in the shade, and rainfall (Tinytag Plus, Gemini Data Loggers Ltd, West Sussex, UK) was recorded in 10 minute intervals at the study site. Wind speed was obtained from the Bureau of Meteorology for the nearest (~7 km) weather station at Wattamolla. We determined the abundance of flying insects over the entire night period with a 12-V ultraviolet insect light twice/week. The light was placed at random locations within two main areas described by the activity of the study animals (~100–1000 m distance); the order was alternated between the areas. Dry weight of insects was determined to the nearest 0.1 mg. We also monitored the flowering of *Banksia* species, identifying species and counting inflorescences along 10 transects (5 × 10 m) once/week.

### Trapping and handling

Animals were captured in box Aluminium traps (Elliott type A, Elliott Scientific Ltd, Upwey, Melbourne, Vic.) baited with a mixture of peanut butter, honey and oats and sprayed with honey water, set overnight in small trees and bushes at a height of 1–2 m. Captured sugar gliders were weighed to the nearest 0.1 g and sexed. Animals were earmarked with a 1 mm ear punch for individual identification and unless implanted with transmitters released on the evening of capture.

### Surgery

We implanted 12 individuals (8 males, 4 females, body mass 125.8 ± 23.9 g) with temperature-sensitive radio transmitters (2 g, Sirtrack, Havelock North, New Zealand) that allowed us to track the individuals and to determine their T_b_. Animals were implanted within two days after capture; all females were non-reproductive at the time of implantation. Transmitters were waxed and calibrated in a water bath to the nearest of 0.1 °C before being implanted intraperitoneally under oxygen/isoflurane anaesthesia through a small abdominal incision^23^. Animals were allowed to recover for one day before being released at the site of capture.

### Radio tracking and measurement of body temperature

After release gliders were radio tracked daily to identify nest trees and monitored for 29–43 days, depending on their capture date. Occasional night-time observations were performed to identify tree hollows, monitor the number of individuals sharing a nest cavity, as well as emergence times. Tree heights and heights of tree hollows were determined using a clinometer (Suunto, Vantaa, Finland). T_b_ was obtained at 10 min intervals with custom-made receiver/loggers^15^ placed close to the nest tree. Large T_b_ variations between activity (T_b_ > 38 °C) and rest phase allowed us to estimate the hours of nightly activity. As observations confirmed that glider never showed a T_b_ above 38 °C before leaving their nest hollow, animals were deemed “active” from the point onwards when either T_b_ increased above 38 °C or the logger failed to record T_b_, because animals moved out of range. Conversely, a decrease in T_b_ below 38 °C and reception of a transmitter signal was associated with inactivity. Animals were considered to be torpid when T_b_ fell below 30 °C (threshold determined by previous studies^15^,^16^). Entry into torpor was defined as the time when T_b_ started decreasing continuously from 34 °C to temperatures below 30 °C. The end point of arousal was defined as the time when T_b_ reached a plateau above 30 °C.

### Calculation of estimated energy savings through torpor use

The energetic significance of daily torpor can be explained by the temperature effect upon tissue metabolic rate and is described by the general equation: ΔT_b_ = 20.1(MR_calc_ + C(T_b_−T_a_)m*SH^24^; whereby ΔT_b_ is the differential between two subsequent T_b_ readings (°C per 10 min), instead of; T_a_ is the daily average of 11.9 °C, C is the thermal conductance (0.069 ml O_2_ °C^−1^ min^−1^ for sugar gliders^20^), m is the body mass of individual study animals (range: 93–149 g), SH is the specific heat of animal tissues (3.47 Jg^−1^°C^−1^) and 1 mlO_2_ is assumed to be equivalent to 20.1 J.

### Data analyses

Data are presented as mean ± 1 standard deviation; *n* denotes the number of individuals, *N* the number of observations. Statistical analyses were conducted in R (R version 3.1.0; 2014-04-10). Normal distribution and homogeneity of variance were tested using Shapiro-Wilk test and Bartlett´s test, respectively. If needed, data were transformed using the Box-Cox function to meet statistical assumptions. Influence of T_a_ on insect availability was tested using a regression analyses. The influence of climate variables (T_a_, average wind speed and rainfall) on nightly activity and TBD was tested using a regression-based approach via generalized linear mixed effects models with “individual” as a random factor. As minimum T_a_ was closely correlated with daily average T_a_ (r^2^ = 0.544, df = 40, p < 0.001; minimum Ta (°C) = 0.632*average Ta (°C) + 1.087), only results for minimum T_a_ are presented. For testing differences in TBD during the storm-night and non-storm nights the analysis was followed by an ANOVA, using individuals as random factor (lme in library nlme; R Core Development Team 2008). The influence of the weather variables on torpor use was tested using a multiple regression analyses. The difference of torpor T_bmin_ during the storm in comparison to all other torpor days was tested using a Wilcoxon rank test. Regression equations are only given for untransformed data.

## Additional Information

**How to cite this article**: Nowack, J. *et al*. Snoozing through the storm: torpor use during a natural disaster. *Sci. Rep*. **5**, 11243; doi: 10.1038/srep11243 (2015).
